# Effects of fluopyram and azadirachtin integration with sunn hemp on nematode communities in zucchini, tomato and sweet potato in Hawaii

**DOI:** 10.21307/jofnem-2021-030

**Published:** 2021-03-06

**Authors:** Philip Waisen, Koon-Hui Wang, Jensen Uyeda, Roxana Y. Myers

**Affiliations:** 1 Department of Plant and Environmental Protection Sciences, University of Hawaii at Manoa, Honolulu, HI, 96822; 2 Department of Tropical Plant and Soil Sciences, University of Hawaii at Manoa, Honolulu, HI, 96822; 3 USDA-ARS Daniel K. Inouye U.S. Pacific Basin Agricultural Research Center, Hilo, HI, 96720

**Keywords:** Chemigation, Free-living nematodes, *Meloidogyne* spp., Nematicides, *Rotylenchulus reniformis*, Soil health

## Abstract

Fluopyram (Velum^®^ One) is a synthetic nematicide and azadirachtin (Molt-X^®^) is a biological nematicide. Both have shown promise against plant-parasitic nematodes on several agriculturally important crops. There is a lack of information on integration of pre-plant sunn hemp (*Crotalaria juncea*) cover crop with these post-plant nematicides, aiming to improve plant-parasitic nematodes management and mitigate any detrimental effects on free-living nematodes. Three field trials were conducted to investigate the effects of fluopyram alone or in combination with pre-plant sunn hemp cover crop, and azadirachtin combined with pre-plant sunn hemp on *Rotylenchulus reniformis* and *Meloidogyne* spp., and free-living nematodes. Zucchini (*Cucurbita pepo*) and tomato (*Solanum lycopersicum*) were grown in Trials I and II, and sweet potato (*Ipomoea batatas*) only was grown in Trial III. In all three trials, early applications of fluopyram at crop planting were effective in suppressing the abundance of *Meloidogyne* spp. (*M. incognita* and *M. javanica*) but it was not effective in reducing *R. reniformis* in the soil. Combining sunn hemp with fluopyram was suppressive to *R. reniformis* on short-term zucchini crop, but not on longer term tomato and sweet potato crops. In addition, application of fluopyram at transplanting was the key to successful suppression of *Meloidogyne* spp. as later fluopyram chemigation (at 2 weeks after planting in Trial II or 1 month after planting in Trial III) had no effect against *Meloidogyne* spp. On the other hand, planting of sunn hemp followed by monthly post-plant azadirachtin application consistently suppressed *R. reniformis*, but this treatment did not suppress *Meloidogyne* spp. Integrating sunn hemp with fluopyram increased zucchini yield by >2.3 folds and that with azadirachtin increased the zucchini yield by >1.7 folds. Although no yield improvement was observed on tomato in Trial II, integrating sunn hemp with azadirachtin and fluopyram increased tomato yield by 0.23 and 1.12 folds, respectively, in Trial I. Marketable yield of sweet potato was increased by 4.5–6.4 folds in all the fluopyram treatments but was only increased 61.5% by sunn hemp plus azadirachtin treatment. While fluopyram alone often reduced the abundance of free-living nematodes, integrating with sunn hemp mitigated the negative impacts of fluopyram on soil health.

Increasing food self-sufficiency is a critical objective for the State of Hawaii that imports 90% of its food from the global market (Department of Business Economic Development and Tourism DBEDT and Hawaii Department of Agriculture HDOA, State of Hawaii, 2012). Currently, the value of vegetables, melons, potatoes (*Solanum tuberosum*), and sweet potatoes (*Ipomoea batatas*) grown by local farmers is $85 million annually plus $6 million in greenhouse tomato sales in Hawaii (United States Department of Agriculture National Agriculture Statistics Service USDA NASS, 2019). Tremendous potential for expansion exists with support for local food production systems. Improving pest and disease management strategies could further bolster yields and stimulate agribusinesses in Hawaii.

Plant-parasitic nematodes are detrimental pests that adversely affect plant health and yields in fruit and vegetable crops. Of particular importance are root-knot (*Meloidogyne* spp.) and reniform (*Rotylenchulus reniformis*) nematodes. On vegetable and tomato crops, nematode infestations result in stunting and poor yields. In a nematode-infested sweet potato field, poor plant growth and the deformity of the tubers are common. Environmental conditions in Hawaii are conducive for year-round nematode growth and reproduction. With limited post-plant nematode management options for disrupting the nematode life cycle, the application of chemical or biological nematicides through chemigation is often needed.

Fluopyram, first marketed as a fungicide and later as a nematicide, is an inhibitor of the succinate dehydrogenase enzyme (Veloukas and Karaoglanidis, 2012). Exposure of *Meloidogyne incognita* and *R. reniformis* to low concentrations of fluopyram resulted in nematode paralysis and reduced penetration of tomato (*Solanum lycopersicum*) roots (Faske and Hurd, 2015). Fluopyram has shown promise against plant-parasitic nematodes on ornamental and agriculturally important crops (Jeschke, 2016; Myers et al., 2020). Applications of fluopyram have shown potential against burrowing nematode (Radopholus similis) by improving anthurium (Anthurium andraeanum) plant vigor and cut flower production (Myers et al., 2020). An in-season application of fluopyram was suppressive to sting nematode (Belonolaimus longicaudatus) on commercial strawberry (Watson et al., 2020). In a tomato pot assay, a single application of fluopyram reduced the second stage juvenile population of M. incognita by 92% six weeks after the treatment (Dahlin et al., 2019). In field trials, tomato yields were increased by 59% following multiple fluopyram applications (Ji et al., 2019). In other field trials on cucurbits, the reduction of *M. hapla* and *M. javanica* population densities by fluopyram was dependent on cropping season in Florida (Khanal and Desaeger, 2020). However, limited information is available on the non-target effects of fluopyram on free-living nematodes.

Azadirachtin is a naturally occurring substance found in seed kernels of neem (*Azadirachta indica*). It is formulated into an emulsifiable concentrate and sold as Molt-X^®^ (BioWorks, Inc., Victor, NY). It is a growth regulator that interferes with molting and metamorphosis in insects (Rembold et al., 2009). This chemical is widely used as a biological insecticide and nematicide because of shared physiological roles such as molting between insects and nematodes. Azadirachtin reduced mobility of *M. incognita* by 36% after a one-day exposure (Lynn et al., 2010). Applications of azadirachtin to greenhouse tomatoes infected with *M. incognita* resulted in a 59% reduction in galls compared to a water control (Myers et al., 2017). Treating okra (*Abelmoschus esculentus*) with azadirachtin at the first sign of galls was shown to be an economical and effective approach to manage *M. incognita* infestation (Khan et al., 2012). Although azadirachtin is a plant-based chemical, there are studies reporting negative impacts on free-living nematodes (Langat et al., 2008) and other beneficial microbial activities (Gopal et al., 2007).

Cultural practices can be implemented to improve soil health and reduce population densities of plant-parasitic nematodes. Planting a sunn hemp (*Crotalaria juncea*) cover crop prior to growing vegetables has been shown to increase the abundance of free-living nematodes among other benefits such as adding organic matter in the soil, and improving soil nutrient cycling (Wang et al., 2003).

This study was guided by the hypotheses that the integration of fluopyram or azadirachtin with pre-plant sunn hemp cover crop would not only be suppressive to plant-parasitic nematodes but also mitigate non-target effects of the nematicides on free-living nematodes. The specific objective of this study was to evaluate the effects of fluopyram treated alone and fluopyram or azadirachtin treated in combination with pre-plant sunn hemp cover crop amendment on plant-parasitic nematodes and free-living nematodes.

## Materials and methods

The hypotheses were tested in three separate field trials, differing to some extent in the number of treatments and the crops grown; the first two with tomato and zucchini and the last one with sweet potato.

### Experiment site

The experiments were conducted at Poamoho Experiment Station, Waialua, on the Island of Oahu, HI (21°32'14.8“N and 158°5'20.3“W). The soil type at the experiment site was a Wahiawa silty clay in the Oxisol order with Tropptic Eutrustox, clayey, kaolinitic, isohyperthermic properties, containing 18.6% sand, 37.7% silt, and 43.7% clay, and soil organic matter of approximately 1.08% in the top 25 cm soil.

### Trial I

In 2017, a field trial was conducted to examine the effects of fluopyram (41.5%, Velum^®^ One, Bayer CropScience, Research Triangle Park, NC) and azadirachtin (3%, Molt-X^®^, BioWorks, Inc., Victor, NY) against root-knot nematodes (*Meloidogyne incognita* and *M. javanica*) and reniform nematode (*Rotylenchulus reniformis*) on either ‘Felix F1’ zucchini (*Cucurbita pepo*) or ‘Komohana’ cherry tomato (*Solanum lycopersicum*). Prior to initiation of the experiment, the field site had a history of soybean (*Glycine max*) crop susceptible to *Meloidogyne* spp. and *R. reniformis*. All soybean residues were tilled into the ground and fifteen 1.2 m × 6.1 m field plots were prepared. Two drip lines, 0.91 m apart, were set up for each plot. One drip line was planted with zucchini and the other one with tomato seedlings. Five treatments were installed including 1) fluopyram applied at 81.8 mL/ha at the time of crop planting (Velum I); 2) fluopyram applied at 81.8 mL/ha at planting and 2 weeks after planting (Velum II); 3) ‘Tropic Sun’ sunn hemp seeded in furrow at 33 kg/ha 2 months prior to crop planting, the generated biomass was incorporated into the soil prior to planting cash crops, and fluopyram was chemigated at 81.8 mL/ha 1 month after planting (SH + Velum); 4) sunn hemp seeded at 33 kg/ha, soil incorporated 2 months after planting and azadirachtin applied monthly at 0.66 mL/ha starting 1 month after planting (SH + MoltX); and 5) an untreated bare ground control. The untreated control treatment and those that were not planted with sunn hemp were maintained bare by spraying herbicide or hand weeding following initial tillage. Fluopyram and azadirachtin were delivered through drip irrigation or chemigation using a Dosatron^®^ Injector (Dosatron International, Inc., Clearwater, FL). Sunn hemp biomass was soil incorporated at 25 Mg/ha in the top 10 cm using a hand-held tiller (American Honda Motor Co., Alpharetta, GA). Each treatment was replicated three times in a randomized complete block design. Three 3-week-old zucchini or 6-week-old tomato seedlings were transplanted at 0.46 m spacing between plants in a row. Fluopyram was chemigated after the field was irrigated to field capacity with volumetric soil moisture of 35.7% and soil temperature of 31–32^°^C. Fluopyram was injected through drip tape (10-cm drip spacing) with a flow rate of 25 mL/min for 60 min equivalent to 8,823 L of water/ha. Azadirachtin was distributed at 6,255 L of water/ha. Chemigation schedules for each trial were shown in Table 1. All plants were fertilized equally using meat and bone meal (9.5–2.5–0.75 N–P–K and 5.0% calcium, Tankage, Baker Commodity Inc., Kapolei, HI) achieving 190 kg N/ha.

**Table 1.** Fluopyram and azadirachtin chemigation schedules for Trials I, II, and III.

	Chemigation schedule		
	Trial I (2017)	Trial II (2018)	Trial III (2019)
Treatments	Zucchini and tomato	Zucchini and tomato	Sweet potato
Velum I	At planting	At planting	At planting and 3 months post-planting
Velum II	At planting and 2 weeks post-planting	At 2 weeks post-planting	At 2 weeks and 3 months post-planting
SH + Velum	At 4 weeks post-planting	At 4 weeks post-planting	At 1 and 3 month(s) post-planting
SH + MoltX	At monthly interval	At monthly interval	At monthly interval
Control	Untreated bare ground	Untreated bare ground	Untreated bare ground

### Trial II

In 2018, a second field trial was superimposed on the first field trial (Trial I) following 3 months of soybean cultivation. All plots were checked for initial population densities of reniform and root-knot nematodes and were found to be not different among plots at the end of the soybean crop prior to soil incorporation. For treatments 3 and 4, sunn hemp cover cropping was planted 2 months before cash crop planting. Soybean or sunn hemp residues were rotor-tilled with a handheld tiller and Trial II was initiated on July 25, 2018 as a repeat of Trial I with a slight modification. While treatments 1, 3, 4, and 5 remained the same as in Trial I, treatment 2 was adjusted to a one-time application of fluopyram at 2 weeks after planting (without the 0-week application as was done in Trial I). Sunn hemp biomass generated in this trial was similar to that in Trial I with an average of 22 Mg/ha.

### Trial III

This trial was conducted in 2019 superimposed on the same field site using sweet potato (*Ipomoea batatas*), a longer-term crop than tomato and zucchini. Prior to initiation of this trial, all plots were tilled and left bare fallow for two months using herbicides. Initial population densities of root-knot and reniform nematodes were checked prior to sunn hemp soil incorporation. For treatments 3 and 4, sunn hemp was grown for two months prior to planting sweet potato. Due to the poor growth of sunn hemp, additional sunn hemp biomass from a nearby field was included to achieve 22 Mg/ha. Rooted sweet potato cuttings were planted at 0.9 m between plants within a row. Since sweet potato is a longer-term crop (6 months growing period), treatments were slightly modified to include second application of fluopyram including 1) fluopyram applied at 81.8 mL/ha at crop planting and 3 months after the crop planting (Velum I); 2) fluopyram applied at 81.8 mL/ha 2 week and 3 months after crop planting (Velum II); 3) soil incorporation of sunn hemp followed by fluopyram applied at 81.8 mL/ha at 1 and 3 month(s) after crop planting (SH + Velum); 4) soil incorporating sunn hemp followed by azadirachtin at 0.66 mL/ha applied monthly beginning 1 month after planting (SH + MoltX); and 5) an untreated control. The chemicals were delivered in the same way as described previously. Sweet potato was fertilized using Suståne^®^ 8-2-4 (Sustane Cooperate, Cannon Falls, MN) to achieve 44.8 kg N/ha.

### Plant growth and yield

Zucchini canopy width and chlorophyll content from the third matured leaf of each plant was measured bi-weekly. Chlorophyll content was measured using a SPAD-502 Chlorophyll Meter (Konica Minolta, Tokyo, Japan 2003). Due to heavy infestation by melon fly (*Bactrocera cucurbitae*), zucchini fruit weight was not recorded, but fruit numbers per plot were recorded weekly from 4 weeks after planting. Tomato was also infested by melon fly, fruits were harvested weekly over 6 weeks starting from 6 weeks after planting, graded to marketable and unmarketable fruits, and weighted. Sweet potato number and weight of marketable and unmarketable roots were recorded 6 months after planting.

### Nematode assay

Initial soil populations of plant-parasitic nematodes were documented at the time of planting sunn hemp cover crop. These data were used for a background check and were not used in the treatment comparison. For treatment comparison, in Trial I, soil samples from zucchini or tomato were collected at 0, 1, and 2 months after crop planting. In Trial II, soil samples were collected at 0 and 2 months after planting zucchini or 0 and 3 months after planting tomato. In Trial III, soil samples were collected at 0, 2, 3 (prior to fluopyram application), and 6 months (at harvest) after sweet potato planting. At each time of sampling, four soil cores per plot were collected from the top 10 cm using a 7.5 cm diameter GroundShark shovel (Forestry Suppliers Inc. Jackson, Mississippi, USA) and composited in a sampling bag. The soil was sifted using a 4-mm^2^ mesh screen and homogenized by handshaking prior to collecting a 250-cm^3^ subsample. Nematodes were extracted from the 250-cm^3^ soil subsample by elutriation and centrifugal flotation (Byrd et al., 1972; Jenkins, 1964). Individual nematodes present in each sample were identified to genus level except for Rhabditidae which was identified only to the family level under Leica^™^ Inverted Microscope (Leica Microsystems Co., Wetzlar, Germany) with reference to Goodey (1963) or Smart and Nguyen (1988). All nematodes identified were grouped into one of the five trophic groups: bacterivores, fungivores, herbivores, omnivores, and predators according to Yeates et al. (1993), and the abundance of each trophic group was enumerated.

At the termination of Trials I and II, individual root systems of zucchini and tomato were uprooted as much as possible using a pitchfork. Roots were weighed and the severity of nematode infestation over the whole root system uprooted was rated based on a 0–10 scale, where 0 = no gall and 10 = plant is dead due to severe galling, according to Bridge and Page (1980).

### Statistical analysis

Data from each field trial were checked for normality using Proc Univariate in SAS version 9.4 (SAS Institute Inc., Cary, NC). Wherever necessary, nematode abundance data were normalized using log10 (x + 1). Nematode abundance data were subjected to a repeated-measures analysis of variance using Proc GLM in SAS. If significant interaction between treatment and sampling time was detected, data were analyzed by sampling time. Zucchini, tomato and sweet potato yield, root weight, and root-gall index, were subjected to a one-way ANOVA. Means were separated using the Waller–Duncan *k*-ratio (*k* = 100) *t*-test whenever appropriate and only true means were presented.

## Results

### Effects on plant-parasitic nematodes

**Figure 1:**
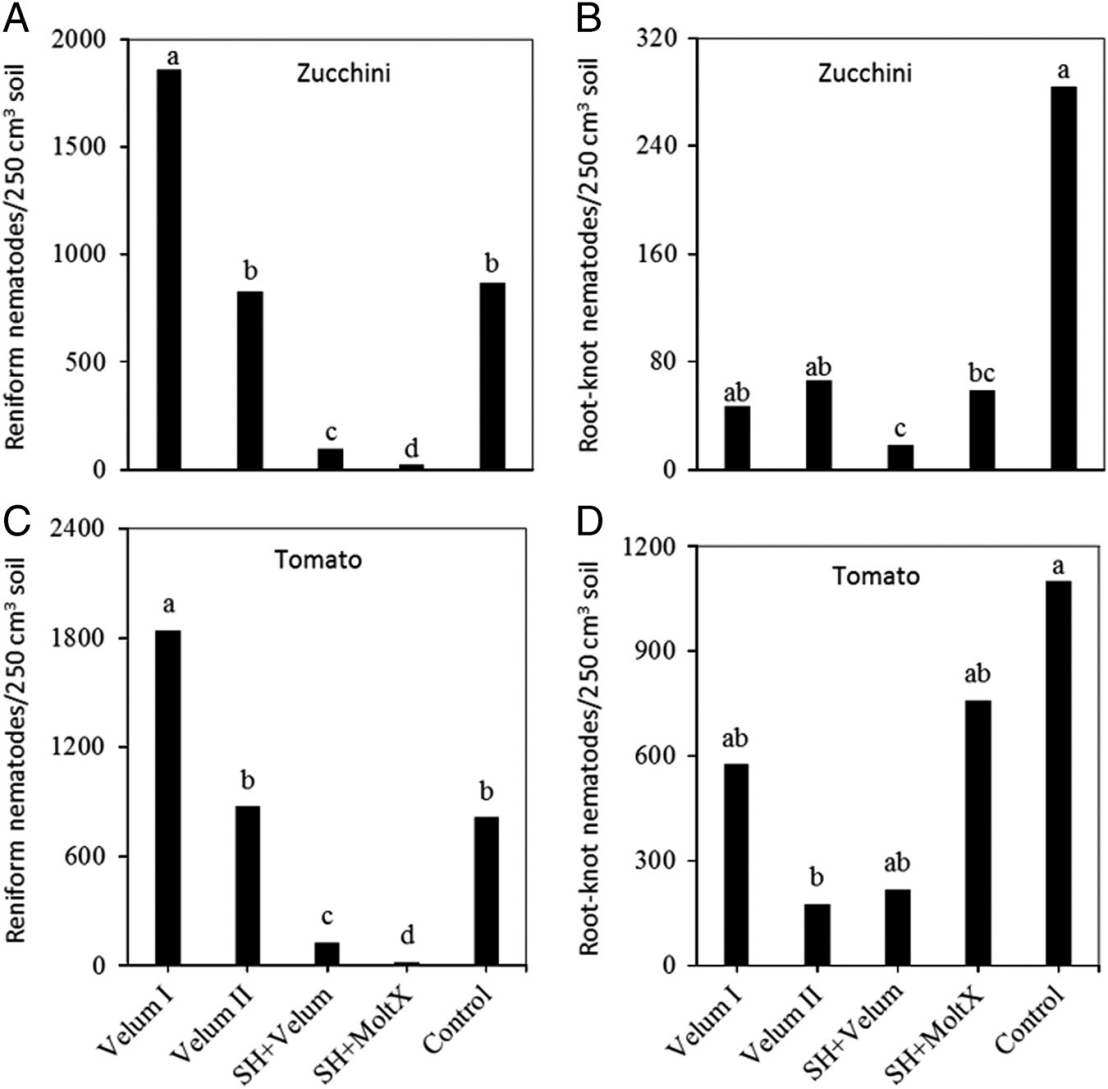
Abundance of A) reniform and B) root-knot nematodes affected by treatments on zucchini or on tomato (C, D) in Trial I. Means (*n*-9) followed by the same letter(s) are not different according to Waller-Duncan *k*-ratio (*k*  =  100) *t*-test. Velum I = fluopyram treatment at crop planting; Velum II  =  fluopyram treatment at crop planting and 2 weeks after the planting; SH + Velum = pre-plant sunn hemp cover crop followed by fluopyram treatment at 1 month after crop planting; SH + MoltX = sunn hemp cover crop and monthly azadirachtin treatment; Control = untreated bare ground.

**Figure 2:**
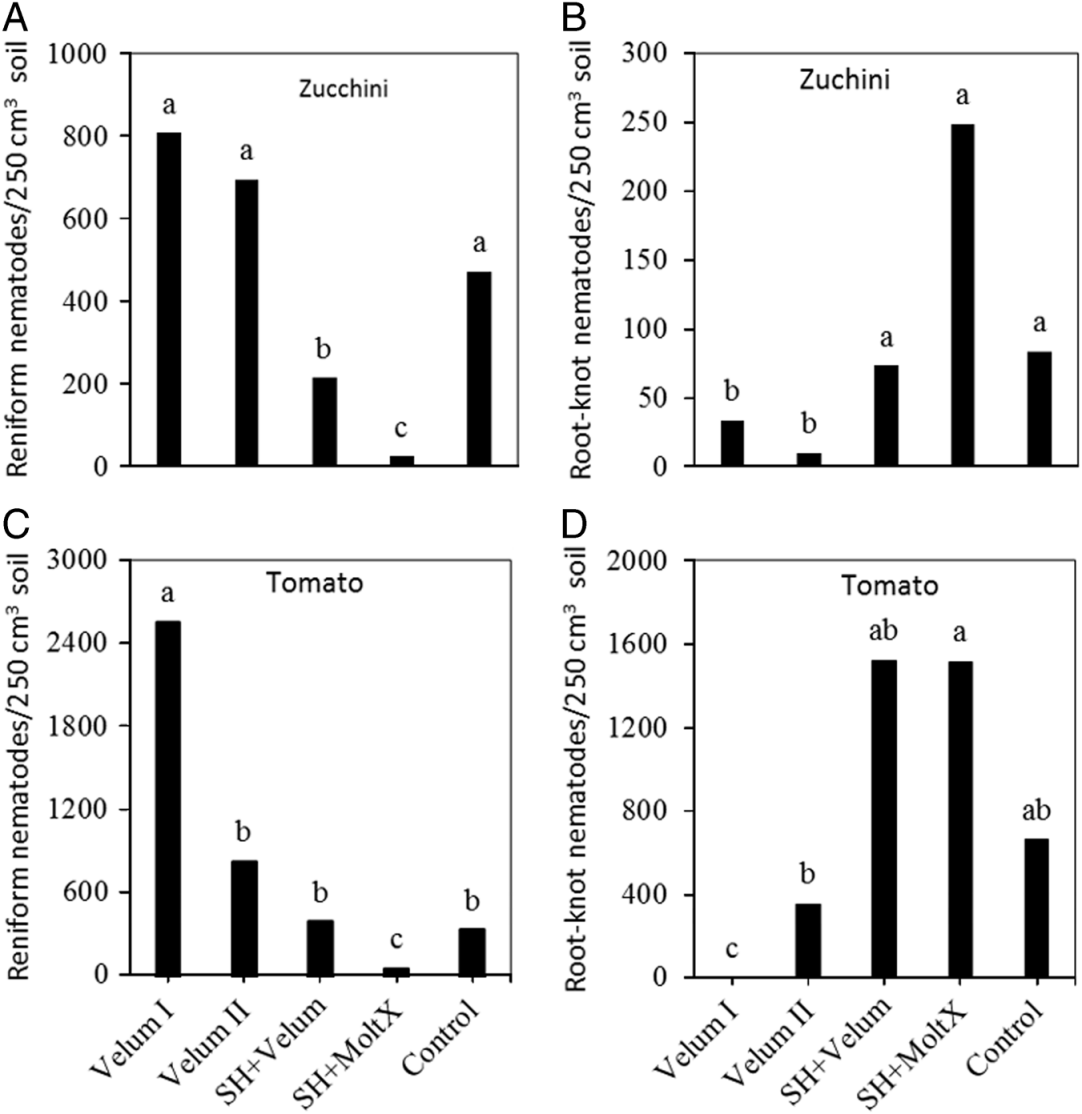
Abundance of A) reniform and B) root-knot nematodes on zucchini or on tomato (C, D) affected by treatments in Trial II. Means (*n* = 6) followed by the same letter(s) are not different according to Waller-Duncan *k*-ratio (*k* = 100) *t*-test. Velum I = fluopyram treatment at crop planting; Velum II = fluopyram treatment at 2 weeks after crop planting; SH + Velum = pre-plant sunn hemp cover crop followed by fluopyram treatment 4 weeks after planting; SH + MoltX = sunn hemp cover crop followed by monthly azadirachtin treatment; Control = untreated bare ground.

No significant difference was detected among treatments on the abundance of *R. reniformis* and *Meloidogyne* spp. at the time of planting sun hemp in Trials I, II, and III. At sunn hemp cover crop planting, average population densities of *R. reniformis* were 202, 153, and 486 per 250 cm^3^ and that of *Meloidogyne* spp. were 15, 7, and 114 per 250 cm^3^ soil in Trials I, II, and III, respectively. There was also no interaction between sampling time and treatment after cash crop planting, thus plant-parasitic nematode abundance data were combined over the two or three sampling dates per crop. On the zucchini, the abundance of *R. reniformis* and *Meloidogyne* spp. in the soil were suppressed by SH + Velum and SH + MoltX compared to the untreated bare ground control in Trial I (*P* < 0.0001; Figure 1A, B). Similarly, SH + Velum and SH + MoltX also suppressed the abundance of *R. reniformis* (Figure 1C) but not that of *Meloidogyne* spp. on tomato. Only Velum II (two-time application of fluopyram) suppressed the abundance of *Meloidogyne* spp. (Figure 1D) compared to the control (*P*  <  0.0001) on tomato plants. None-the-less, one or two-time applications of fluopyram (Velum I or II) was not different from each other in terms of suppressing *Meloidogyne* or *R. reniformis* in this trial. Thus, in Trial II, a slight modification was done to compare one-time application of fluopyram at crop planting (Velum I) to fluopyram at 2 weeks after the planting (Velum II). On zucchini, SH + Velum and SH + MoltX were still the only treatments that suppressed the abundance of *R. reniformis* (Figure 2A), but this time Velum I and Velum II suppressed the abundance of *Meloidogyne* spp. compared to the control but not SH + Velum and SH + MoltX (Figure 2B). On the other hand, 3 months after planting of the tomato, SH + MoltX was the only treatment that suppressed the soil population of *R. reniformis* (Figure 2C) on tomato, but only Velum I (applying fluopyram at planting) suppressed the abundance of *Meloidogyne* spp. (Figure 2D) compared to the control (*P*  <  0.004). Applying fluopyram at 2 weeks after planting (Velum II) no longer suppressed the abundance of *Meloidogyne* spp. in the soil at the end of the tomato crop (3 months after planting).

**Figure 3:**
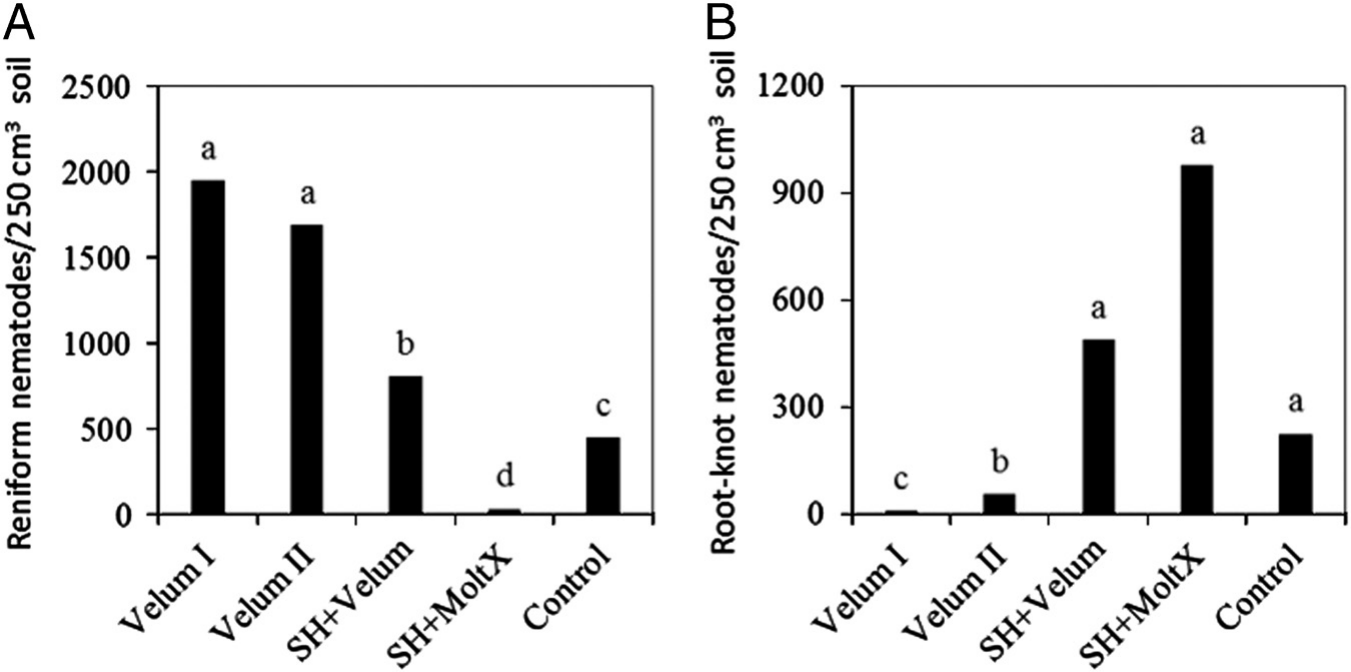
Abundance of A) reniform and B) root-knot nematodes on sweet potato affected by treatments in Trial III. Means (*n* = 12) followed by the same letter(s) are not different according to Waller-Duncan *k*-ratio (*k* = 100) *t*-test. Velum I = fluopyram treatment at 0 and 3 months after crop planting; Velum II = fluopyram treatment at 2 weeks and 3 months after crop planting; SH + Velum = pre-plant sunn hemp cover crop followed by fluopyram at 1 and 3 months after crop planting; SH + MoltX = pre-plant sunn hemp cover crop and monthly azadirachtin treatment; Control = untreated bare ground.

In Trial III, there was no significant difference in population densities of root-knot and reniform nematodes among treatments at sweet potato planting, and no significant interaction between sampling dates and treatments were detected, thus nematode abundance data were combined over the three sampling dates after sweet potato planting (at 2, 3, and 6 months after planting). Similar to results in Trial I and Trial II, SH + MoltX suppressed the soil population of *R. reniformis* (Figure 3A) and Velum I and Velum II suppressed soil populations of *Meloidogyne* spp. (Figure 3B) compared to the control (*P  <  *0.0001). Applying fluopyram at planting (Velum I) again suppressed *Meloidogyne* spp. better than applying it at a later planting date (Velum II, 2 weeks after planting). It is worth mentioning that throughout all three trials, the abundance of *R. reniformis* in the soil was increased in fluopyram treatments (Velum I or sometimes Velum II) compared to the control (Figures 1A, C, 2C, and 3A, C). We did not have root staining data to examine if a higher infection rate of roots by *R. reniformis* was associated with higher numbers of reniform nematodes in the soil.

### Effects of treatments on root-gall index, plant growth and yield

In Trial I, zucchini chlorophyll content, canopy width, root weight and fruit numbers were all ranked higher for SH + Velum and SH + MoltX followed by Velum I and II (Table 2). Control treatment resulted in lower chlorophyll content and fruit number/plant than Velum II, SH + Velum and SH + MoltX (*P* < 0.0001) and lower canopy width than all other treatments (*P* < 0.0001) in Trial I. Zucchini was terminated at 2 months after planting with relatively low *Meloidogyne* pressure ( < 300 nematodes/250 cm^3^ soil), thus no significant difference in root-gall indices (RGI) among treatments. Nonetheless, Velum I had the numerically lowest RGI whereas the control had the highest RGI on zucchini. Tomato was terminated at 3 months after planting, with much higher *Meloidogyne* pressure (>1,000 nematodes/250 cm^3^ soil). A significant difference in RGI was observed among treatments on tomato in Trial I where all treatments with fluopyram (Velum I, II, and SH + Velum) resulted in lower RGI than the control (*P* < 0.012) but SH + MoltX failed to reduce RGI on tomatoes. Larger RGI also resulted in a heavier root weight of tomato than all the fluopyram treatments (*P* < 0.020). Despite complication in tomato yield due to fruit flies infestation, Velum I and SH + MoltX had higher tomato yield (combined marketable and unmarketable) than the control (*P* <  0.0077). Velum II had a lower tomato yield than the control (*P* < 0.0077).

**Table 2.** Chlorophyll content, canopy width, fruit number, and *Meloidogyne*-induced root-gall index on zucchini and tomato affected by treatments in Trials I and II.

Treatment	Chlorophyll content (SPAD)		Canopy width (cm)		Root weight (g)		Fruit no./plant		Root-gall index (0-10 scale)	
(Trial)	I	II	I	II	I	II	I	II	I	II
*Zucchini*										
Velum I	36 ± 1bc^z^	47 ± 2a	87 ± 7b	114 ± 5c	89 ± 12b	35 ± 4b	50 ± 3bc	7 ± 1ab	3 ± 1a	2 ± 0c
Velum II	37 ± 1b	46 ± 1a	89 ± 8b	118 ± 6bc	102 ± 27ab	31 ± 4b	40 ± 5c	5 ± 1bc	3 ± 1a	2 ± 1bc
SH + Velum	44 ± 1a	47 ± 1a	117 ± 4a	134 ± 3a	155 ± 12a	49 ± 4a	72 ± 3a	10 ± 1a	4 ± 1a	3 ± 1b
SH + MoltX	42 ± 2a	45 ± 1a	109 ± 6a	127 ± 6ab	124 ± 19ab	39 ± 4ab	54 ± 5b	6 ± 1bc	4 ± 2a	5 ± 2a
Control	34 ± 2c	44 ± 1a	69 ± 10c	98 ± 6d	71 ± 40b	35 ± 4b	20 ± 11d	3 ± 1c	7 ± 0a	5 ± 2a
*Tomato*										
	Chlorophyll content (SPAD)		Canopy width (cm)		Root weight (g)		Fruit weight g/plot		Root-gall index (0-10 scale)	
Velum I	–	44 ± 1a	–	–	110 ± 10bc	53 ± 9a	3525 ± 717a	123 ± 50a	4 ± 1b	2 ± 1c
Velum II	–	43 ± 1ab	–	–	95 ± 21c	76 ± 17a	1074 ± 471c	101 ± 29a	3 ± 1b	4 ± 1b
SH + Velum	–	45 ± 1a	–	–	92 ± 3c	87 ± 22a	1995 ± 258b	86 ± 32a	2 ± 1b	6 ± 1ab
SH + MoltX	–	40 ± 1b	–	–	153 ± 11ab	64 ± 19a	3460 ± 80a	105 ± 45a	6 ± 0ab	7 ± 1a
Control	–	43 ± 2ab	–	–	164 ± 26a	77 ± 17a	1625 ± 604b	112 ± 65a	8 ± 0a	8 ± 0a

Note: ^z^Means ± standard error in a column followed by the same letter(s) are not different, according to the Waller–Duncan *k*-ratio (*k* = 100) *t*-test.

In Trial II, similar results were observed on both zucchini and tomato where Velum I resulted in the lowest RGI, followed by lower RGI in Velum II and SH + Velum than in control and SH + MoltX (*P < *0.0001, Table 2). This was reflected in higher zucchini fruit numbers for SH + Velum and Velum I than the control (*P < *0.0013). Regardless of the RGI, treatments with sunn hemp green manure tended to increase canopy width compared to the control on zucchini (*P < *0.0001). Whereas despite the differences in RGI among treatments on tomatoes, all treatments did not affect chlorophyll content, root weight nor fruit weight on tomatoes (Table 2).

In Trial III, where the treatment effects were evaluated over a longer period (6 months) and measurement of RGI or canopy width was difficult on sweet potato, the marketable swollen root number or weight were better indicators of the plant response to the soil treatments. The result showed that all fluopyram treatments yielded higher marketable sweet potato number (*P* < 0.0082) and weight (*P < *0.0003) than SH + MoltX and Control (Table 3), following the opposite trend of the *Meloidogyne* abundance in the soil rather than that of *R. reniformis* (Figure 3).

**Table 3.** Marketable and unmarketable sweet potato root number and weight affected by treatments in Trial III.

	Marketable		Unmarketable	
Treatment	Number	Weight (kg)	Number	Weight (kg)
Velum I	2 ± 0a^z^	0.7 ± 0.2a	2 ± 1a	0.4 ± 0.1a
Velum II	2 ± 0a	0.8 ± 0.4a	2 ± 1a	0.3 ± 0.1a
SH + Velum	3 ± 1a	1.0 ± 0.3a	3 ± 1a	0.7 ± 0.2a
SH + MoltX	1 ± 0b	0.2 ± 0.1a	2 ± 0a	0.7 ± 0.3a
Control	0 ± 0b	0.1 ± 0.1b	2 ± 0a	0.5 ± 0.1a

^z^Means ± standard error (*n* = 3) in a column followed by the same letter(s) are not different according to the Waller–Duncan *k*-ratio (*k* = 100) *t*-test.

### Effects on free-living nematodes

In Trial I, population densities of each free-living nematode trophic group prior to crop planting were not different among treatments, thus these data were not presented. This allowed a fair comparison among treatments. There was no interaction between sampling time and treatment for the zucchini trial, thus nematode abundance data were presented from the average of 2 sampling dates (Table 4). Minimal to no predatory nematodes were detected in this field, thus their numbers were not presented. Both Velum I and II resulted in lower abundance (*P* < 0.0037) of bacterivorous and fungivorous nematodes than SH + Velum and SH + MoltX, though all treatments were not different from the control. In addition, no difference in the abundance of omnivorous nematodes was observed among treatments (Table 4). In the same trial, abundance of free-living nematodes in the tomato plots showed a significant interaction between sampling date and treatment effects, thus data were presented by sampling date. At 2 months after planting, the abundance of bacterivores was increased by SH + Velum, but Velum I reduced the abundance of this nematode compared to the control (*P* < 0.0023, Table 5). At the same time, SH + Velum and SH + MoltX increased fungivores (*P* < 0.0002) while only SH + MoltX increased the abundance of omnivores (*P* < 0.0224, Table 3). Enhancement of the abundance of bacterivorous nematodes by SH + MoltX and suppression of bacterivorous nematodes by Velum I and II persisted for 3 months after tomato planting (*P* < 0.0004, Table 5). At the same time, the abundance of omnivores was reduced by Velum I, II and SH + Velum treatments compared to the control (*P* < 0.0005, Table 5).

**Table 4.** Abundance (/250 cm^3^ of soil) of bacterivorous, fungivorous, and omnivorous nematodes on zucchini affected by treatments Trial I.

Treatments	Bacterivores	Fungivores	Omnivores
Velum I	614 ± 178b^z^	392 ± 226c	21 ± 18a
Velum II	827 ± 489b	360 ± 208c	30 ± 21a
SH + Velum	1203 ± 217a	722 ± 417ab	23 ± 16a
SH + MoltX	1538 ± 682a	844 ± 488a	40 ± 28a
Control	871 ± 195ab	416 ± 150bc	23 ± 13a

^z^Means ± standard error (*n* = 9) are data collected at 0, 4, and 8 weeks after zucchini planting. Means in a column followed by the same letter(s) are not different, according to the Waller–Duncan *k*-ratio (*k* = 100) *t*-test.

**Table 5.** Abundance of bacterivorous, fungivorous, and omnivorous nematodes (/250 cm^3^ of soil) on tomato affected by treatments in Trial I.

Treatments	Bacterivores	Fungivores	Omnivores
*2 months after planting*			
Velum I	149 ± 54d	253 ± 61b	3 ± 3b
Velum II	393 ± 90cd	410 ± 80b	7 ± 3b
SH + Velum	2193 ± 769a	900 ± 120a	40 ± 30ab
SH + MoltX	1623 ± 613ab	1123 ± 185a	220 ± 157a
Control	587 ± 148bc	293 ± 18b	10 ± 6b
*3 months after planting*			
Velum I	140 ± 36c	103 ± 60b	0 ± 0b
Velum II	70 ± 25d	127 ± 15b	0 ± 0b
SH + Velum	320 ± 76b	407 ± 235a	0 ± 0b
SH + MoltX	817 ± 147a	407 ± 235a	57 ± 14a
Control	370 ± 35b	180 ± 104ab	47 ± 27a

^z^Means ± standard error (*n* = 3) followed by the same letter(s) at each sampling time are not different according to the Waller–Duncan *k*-ratio (*k* = 100) *t*-test.

Trial II was superimposed on the field site at Trial I, thus population densities of bacterivorous nematodes at planting were continued to be higher in SH + Velum and SH + MoltX than Velum I, II and the control (*P* < 0.0035, Table 6). However, no difference in the abundance of fungivorous and omnivorous nematodes was detected at cash crop planting. At the termination of the zucchini crop in Trial II, though no difference in the abundance of bacterivores among treatments was observed, SH + Velum and SH + MoltX increased fungivores number compared to Velum I, II and control (*P* < 0.0045, Table 6). Although no significant difference in the abundance of omnivorous nematodes was detected, Velum I and II resulted in zero number of omnivorous nematodes in the zucchini plots (Table 6). In terms of free-living nematode abundance in tomato plots of Trial II taken at 3 months after planting (at the termination of tomato crop), more apparent effects of SH + Velum and SH + MoltX in increasing the abundance of bacterivores and fungivores as compared to the control (*P* < 0.0048, Table 7). Unfortunately, continuous practice of Velum I and II chemigation not only reduced bacterivorous but also omnivorous nematodes (*P* < 0.0046) compared to the control. Interestingly, planting and incorporating sunn hemp residues into the soil prior to fluopyram treatment eliminated this negative impact on potential indicators of soil health.

**Table 6.** Abundance (/250 cm^3^ of soil) of bacterivorous, fungivorous, and omnivorous nematodes on zucchini affected by treatments in Trial II.

	Bacterivores		Fungivores	Omnivores
Treatments	0 week	8 weeks		
Velum I	40 ± 31b^z^	353 ± 175a	118 ± 41b	0 ± 0a
Velum II	50 ± 25b	194 ± 95a	95 ± 37b	0 ± 0a
SH + Velum	547 ± 115a	510 ± 216a	450 ± 96a	8 ± 5a
SH + MoltX	470 ± 118a	547 ± 235a	553 ± 113a	13 ± 6a
Control	53 ± 15b	633 ± 111a	87 ± 47b	18 ± 13a

**Table 7.** Abundance (/250 cm^3^ of soil) of bacterivorous, fungivorous, and omnivorous nematodes on tomato affected by treatments in Trial II.

Treatments	Bacterivores	Fungivores	Omnivores
Velum I	68 ± 19c^z^	90 ± 16b	7 ± 7bc
Velum II	110 ± 35bc	77 ± 22b	5 ± 5c
SH + Velum	702 ± 122a	563 ± 158a	35 ± 27ab
SH + MoltX	1162 ± 457a	657 ± 194a	62 ± 30a
Control	163 ± 65b	62 ± 17b	43 ± 25a

^z^Means ± standard error (*n* = 6) are data collected at 0 and 12 weeks after tomato planting. Means in a column followed by the same letter(s) are not different according to the Waller–Duncan *k*-ratio (*k* = 100) *t*-test.

In Trial III where a longer-term impact of soil treatments on free-living nematodes was examined on sweet potato, a similar trend was observed. The impact of Velum I and II on free-living nematodes did not dissipate even after 7 months of crop growth. The abundance of bacterivores was reduced at 2 (*P < *0.0063), 3 (*P < *0.0177), and 7 (*P < *0.0023) months after treatment (Table 8). While the effect of SH + Velum and SH + MoltX on the abundance of bacterivores was not significant compared to the control in sweet potato, these treatments increased the abundance of fungivores compared to the control (*P < *0.0001, Table 8). Although the abundance of omnivores in SH + Velum was not different from the control, it was lower than that in the SH + MoltX (*P < *0.0016).

**Table 8.** Abundance (/250 cm^3^ of soil) of bacterivorous nematodes on sweet potato affected by treatments in Trial III.

	Bacterivores					
Treatments	0 week	2 months	3 months	7 months	Fungivores	Omnivores
Velum I	270 ± 91ab^z^	313 ± 55c	137 ± 47bc	43 ± 20b	83 ± 29b	1 ± 1c
Velum II	310 ± 95ab	690 ± 316bc	117 ± 47c	70 ± 12b	113 ± 32b	4 ± 4c
SH + Velum	617 ± 292ab	723 ± 20ab	293 ± 48abc	280 ± 86a	693 ± 339a	6 ± 3bc
SH + MoltX	820 ± 354a	1520 ± 525a	803 ± 158a	563 ± 206a	460 ± 207a	23 ± 8a
Control	150 ± 111b	1223 ± 308a	350 ± 66ab	390 ± 83a	57 ± 12b	19 ± 8ab

^z^Means ± standard error (*n* = 3 for bacterivores or *n* = 12 for fungivores and omnivores). Means in a column followed by the same letter(s) are not different, according to the Waller–Duncan *k*-ratio (*k* = 100) *t*-test.

## Discussion

### Effects on plant-parasitic nematodes

In general, results from all three trials demonstrated that early application of fluopyram at crop planting was key to effective suppression of *Meloidogyne* spp. abundance, but this was not effective in suppressing *R. reniformis* in all three trials. These results are in line with previous nematode motility assays where fluopyram provided more nematostatic effect on *Meloidogyne* spp. than *R. reniformis* (Faske and Hurd, 2015). Although low concentrations of fluopyram were found to be effective at inhibiting infection of tomato roots by *R. reniformis* (Faske and Hurd, 2015), reproduction of *R. reniformis* was not suppressed as reflected on high soil populations of *R. reniformis* in this study. However, combining pre-plant sunn hemp cover crop with fluopyram (SH + Velum) was able to suppress *R. reniformis* on a short-term crop like zucchini, but not on a longer-term crop like tomato or sweet potato. Sunn hemp is known to provide allelopathic effects when soil incorporated against plant-parasitic nematodes including *R. reniformis* (Wang et al., 2001). Thus, fluopyram in combination with a sunn hemp treatment could have a synergistic effect against *R. reniformis,* whereby sunn hemp’s allelopathic compound could have reduced the population densities of *R. reniformis* at crop planting, allowing post-plant chemigation of fluopyram to inhibit further infection of crop roots by *R. reniformis.* This suppressive effect did not last more than two months after fluopyram injection, thus failure of SH + Velum to reduce *R. reniformis* numbers at tomato harvest (>2 months after treatment) or at sweet potato harvest (3 months after last fluopyram treatment).

The suppression of *Meloidogyne* spp. by fluopyram more than *R. reniformis* could be partly explained by possible resource competition between *R. reniformis* and *Meloidogyne* spp. *Meloidogyne incognita* and *R. reniformis* are known to antagonize one another when initial inoculum ratio of one species is higher than the other. In concomitant infections, *M. incognita* was susceptible to the antagonistic effect of *R. reniformis* (Diez et al., 2003). In this study, *R. reniformis* population densities were higher than that of *Meloidogyne* spp. in the three field trials.

The suppressive activity of fluopyram alone on *Meloidogyne* spp. in this study was in line with previous findings where it inhibited infection of *M. incognita* on tomato roots (Dahlin et al., 2019; Faske and Hurd, 2015; Ji et al., 2019), reduced severity of *M. incognita*–induced root galling on cucumbers (*Cucumis sativus*) (Hajihassani et al., 2019), and showed potential for managing *M. incognita* on lima bean, *Phaseoulus lunatus* (Jones et al., 2017). Faske and Brown (2019) showed that the greatest effect of fluopyram on *M. incognita* mortality was up to 10 cm deep in sandy soil. Li et al. (2020) then reported that fluopyram was most effective against *Meloidogyne* spp. at concentration of 60 g·ha^−1^ a.i. applied through 200 L·ha^−1^ of irrigation water at 2 L·h^−1^ flow velocity by chemigation on eggplant (*Solanum melongena*).

The current study demonstrated that application of fluopyram at planting is the key to the success of suppressing *Meloidogyne* spp. This is because delayed chemigation of fluopyram to 2 weeks after planting did not achieve the same suppression against root-knot nematodes as the application at crop planting. In Trial III, where fluopyram was chemigated at 2 weeks and 3 months (Velum II) after sweet potato planting, though it still suppressed the abundance of *Meloidogyne* spp. compared to the control, its effect was lower than that achieved by fluopyram chemigated at 0 and 3 months (Velum I) after planting.

On the other hand, planting of sunn hemp cover crop followed by monthly post-plant azadirachtin application (SH + MoltX) suppressed *R. reniformis* consistently but this treatment was not suppressive to *Meloidogyne* spp. Although leaf extracts and amending soil with oil cake of neem (*Azadirachta indica*) were shown to be effective against *Meloidogyne* spp. (Javed et al., 2008; Saravanapriya and Sivakumar, 2005), effects of azadirachtin appeared to be dependent on concentration and life stages of *Meloidogyne* spp. In laboratory trials, azadirachtin at 0.1% did not affect hatching of *M. javanica* (Javed et al., 2008), but at 0.001% it immobilized *M. incognita* juveniles, and at 17% (NeemAzal^®^-U) it decreased hatching and viability of *M. incognita* in pot trials (Meyer et al., 2012). In this study, monthly chemigation of 3% azadirachtin was only initiated 1 month after planting and did not suppress *Meloidogyne* spp. but did suppress *R. reniformis* consistently in all three trials. This might have been due to the longer life cycle of *R. reniformis* (J2, J3 and male) in the soil compared to *Meloidogyne* spp. (only J2). Thus, *R. reniformis* might have longer exposure to azadirachtin in the soil.

Regardless of differential effects of azadirachtin and fluopyram on different species of plant-parasitic nematodes, green manure effect of sunn hemp in SH + Velum increased zucchini yield by 2.6 and 2.3 folds in Trials I and II, respectively; and SH + MoltX increased zucchini yield by 1.7 and 2.0 folds in Trials I and II, respectively. No yield improvement was observed on tomato on Trial II, but SH + MoltX and SH + Velum increased tomato yield by 22.8 and 112%, respectively, in Trial I. On the contrary, *Meloidogyne* appeared to be the more damaging plant-parasitic nematode on sweet potato than *R. reniformis* and thus marketable yield of sweet potato was increased 4.5 to 6.4 folds by any of the Velum treatments (Velum I, II, or SH + Velum) but only 61.5% increased by SH + MoltX despite effective suppression of *R. reniformis* by the later treatment.

### Effects on free-living nematodes

Unfortunately, fluopyram imposed some negative effect on the abundance of free-living nematodes in particular on longer-term crops (tomato and sweet potato). Interestingly this negative impact of fluopyram was significantly mitigated by integration with sunn hemp cover cropping. In fact, SH + Velum either increased the abundance of bacterivores or fungivores or both on tomato and sweet potato as compared to the control. Whereas SH + MoltX tended to always increase bacterivorous, fungivorous or omnivorous nematodes. Disturbance of free-living nematodes by other non-fumigant types of nematicides has long been studied (Adams et al., 1979; Simpkin and Coles, 1981). Only limited studies have documented the effects of fluopyram on free-living nematodes. Grabau et al. (2020) reported no effect of fluopyram on any free-living nematodes on peanuts (*Arachis hypogaea*), but Waldo et al. (2019) found fluopyram had a substantial negative impact on free-living nematodes in bermudagrass (*Cynodon* spp.). Our findings are in line with that of Waldo et al. (2019) that fluopyram alone would suppress free-living nematodes including bacterivores, fungivores and omnivores that are important indicators of soil health. Although Achook^®^ (0.03% azadirachtin) alone also reduced free-living nematode abundance (Langat et al., 2008), current results here showed that integrating a cover crop such as sunn hemp prior to fluopyram or azadirachtin treatment can mitigate these negative effects of nematicide on free-living nematodes. This is consistent with previous reports that integrating sunn hemp cover cropping could mitigate the negative impact of solarization on soil health (Marahatta et al., 2012).

This study demonstrated that *Meloidogyne* spp. and *R. reniformis* can be managed better using fluopyram and azadirachtin, respectively. Treating fluopyram alone can compromise soil health but integrating with pre-plant sunn hemp cover crop amendment can ameliorate its non-target effects on soil health. Future study needs to investigate whether integration of fluopyram and azadirachtin chemigation along with pre-plant sunn hemp cover crop amendment could concurrently manage *Meloidogyne* spp. and *R. reniformis* as well as improving soil health.
